# Using Transcription Modules to Identify Expression Clusters Perturbed in Williams-Beuren Syndrome

**DOI:** 10.1371/journal.pcbi.1001054

**Published:** 2011-01-20

**Authors:** Charlotte N. Henrichsen, Gábor Csárdi, Marie-Thérèse Zabot, Carmela Fusco, Sven Bergmann, Giuseppe Merla, Alexandre Reymond

**Affiliations:** 1 The Center for Integrative Genomics, University of Lausanne, Lausanne, Switzerland; 2 Department of Medical Genetics, University of Lausanne, Lausanne, Switzerland; 3 Swiss Institute of Bioinformatics, Lausanne, Switzerland; 4 Centre de Biotechnologie Cellulaire, Hospices Civils de Lyon, Groupement Hospitalier Est, Bron, France; 5 Laboratory of Medical Genetics, IRCCS- Casa Sollievo della Sofferenza, San Giovanni Rotondo, Italy; Tel Aviv University, Israel

## Abstract

The genetic dissection of the phenotypes associated with Williams-Beuren Syndrome (WBS) is advancing thanks to the study of individuals carrying typical or atypical structural rearrangements, as well as *in vitro* and animal studies. However, little is known about the global dysregulations caused by the WBS deletion. We profiled the transcriptomes of skin fibroblasts from WBS patients and compared them to matched controls. We identified 868 differentially expressed genes that were significantly enriched in extracellular matrix genes, major histocompatibility complex (MHC) genes, as well as genes in which the products localize to the postsynaptic membrane. We then used public expression datasets from human fibroblasts to establish transcription modules, sets of genes coexpressed in this cell type. We identified those sets in which the average gene expression was altered in WBS samples. Dysregulated modules are often interconnected and share multiple common genes, suggesting that intricate regulatory networks connected by a few central genes are disturbed in WBS. This modular approach increases the power to identify pathways dysregulated in WBS patients, thus providing a testable set of additional candidates for genes and their interactions that modulate the WBS phenotypes.

## Author Summary

A fundamental question in current biomedical research is to establish a link between genomic variation and phenotypic differences, which encompasses both the seemingly neutral diversity, as well as the pathological variation that causes or predisposes to disease. Once the primary genetic cause(s) of a disease or phenotype has been identified, we need to understand the biochemical consequences of such variants that eventually lead to increased disease risk. Such phenotypic effects of genetic differences are supposedly brought about by changes in expression levels, either of the genes affected by the genetic change or indirectly through position effects. Thus, transcriptome analyses seem appropriate proxies to study the consequences of structural variation, such as the 7q11.23 deletion present in individuals with Williams-Beuren syndrome (WBS). Here, we present an approach that takes experimental data into account instead of relying solely on functional annotation, following the rationale that coherently regulated genes are likely to play a role in the same biological process. While our algorithm can be applied to expression data from any source, our study provides a resource for the identification of additional candidate genes and pathways to explain the WBS phenotype, as well as a basis for uncovering novel functional interactions between sets of genes.

## Introduction

Williams-Beuren Syndrome (WBS; OMIM #194050) is a *de novo* neurodevelopmental disorder occurring in approximately 1/10'000 births. WBS is characterized by mental retardation, with a unique cognitive and personality profile. Clinical features include supravalvular aortic stenosis (SVAS), connective tissue anomalies, distinctive facial features (elfin face), short stature, hypertension, infantile hypercalcemia, dental, kidney and thyroid abnormalities, premature ageing of the skin, elevated body fat percentage, impaired glucose tolerance and silent diabetes. The cognitive hallmark of the condition is a striking contrast between a relative strength in auditory memory and language abilities, and a profound impairment in visuospatial construction. WBS individuals are hypersensitive to sound, with strong emotional responses to music, either positive or negative, and some individuals display unusual musical skills. In addition to this hyperacusis, which is thought to be due to the absence of acoustic reflexes, WBS individuals may suffer from sensorineural hearing loss as they age. They are also very sociable, emphatic, loquacious and over-friendly, with a complete lack of fear towards strangers. Many present an attention deficit disorder with hyperactivity and anxiety [1]–[9].

The WBS is associated with a microdeletion within the 7q11.23 chromosomal band, which encompasses 28 genes [10]–[13]. It is flanked by specific low copy repeats that serve as substrate for non-allelic homologous recombination leading to the deletion [14]. These rearrangements are facilitated by the paracentric inversion of the region [14], [15], as well as the presence of a specific copy number variant [16]. The most common deletion, occurring in approximately 95% of cases, involves a 1.5 megabase (Mb) segment, while a larger 1.84 Mb deletion is observed in about 1 of 20 cases [14], [17]. Larger and smaller atypical deletions have been reported in sporadic cases [18]–[31].

While the primary cause of WBS is well-understood, we still know little about the molecular basis of the phenotype. Only very recently, strains of mice were engineered to carry complementary half-deletions of the region syntenic to the WBS region, which replicate several features of WBS, including abnormal social interaction phenotypes [32]. Yet, so far the dissection of the phenotype relies mainly on evidence from other mouse models — e.g. single gene knock-out — and atypical deletions in humans. Findings from these studies suggest some correlations between hemizygosity of certain genes and specific phenotypic features seen in WBS individuals. For example, the SVAS phenotype was shown to be unequivocally associated with haploinsufficiency of the elastin gene [33]–[35]. Furthermore, mouse models hemizygote for some of the orthologs of the WBS deletion most telomerically-mapping genes suggested that these were linked to craniofacial abnormalities (*GTF2I* and *GTF2IRD1* genes) [36], tooth anomalies and visuospatial deficit (*GTF2I*, *GTF2IRD1* and *GTF2IRD2* genes) [22], [37], as well as deficits in motor coordination (*CLIP2*) [38]. Likewise, the function of the carbohydrate response element-binding protein (*MLXIPL*, a.k.a. *ChREBP* or *WBSCR14*) in the regulation of the expression of enzymes involved in glucose and lipid metabolism [39]-[43] suggests that its haploinsufficiency is associated with the higher relative body fat, silent diabetes and/or impaired glucose tolerance found in adult WBS individuals [2].

We showed in previous work that the vast majority of the genes hemizygous due to the 7q11.23 deletion are underexpressed in lymphoblastoid cell lines and fibroblasts derived from patients [44], consistent with their possible role in some of the WBS phenotypes. Some of the genes that map to the flank of the microdeletion might also influence the WBS phenotype, as it was recently shown that structural rearrangements affect the relative expression levels of neighboring normal-copy genes ([44]–[48], reviewed in [49], [50]). To identify which downstream pathways are perturbed in WBS by these two classes of human chromosome 7 (HSA7) genes, we generated genome-wide transcription profiles for primary fibroblasts from eight individuals with WBS and nine sex- and age-matched controls. We first focus on differentially expressed genes and then on co-expressed gene sets to elucidate the genes and pathways that are dysregulated in WBS and how they may contribute to its clinical phenotypes.

## Results

### Classical single gene analysis and its limitations

#### Differentially expressed genes

**Figure 1:**
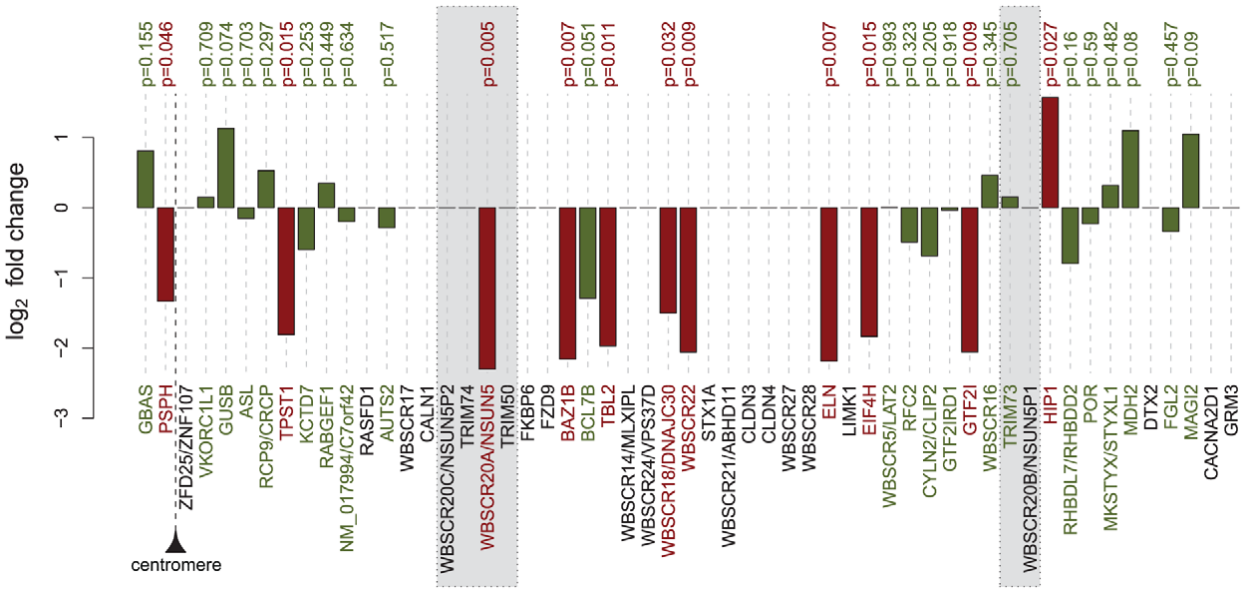
Differential expression of the WBS hemizygous and flanking genes. Genes are ordered according to their chromosomal position. Shaded areas represent the LCRs flanking the deletion. Gene names are indicated at the bottom and corresponding differential expression *P*-values at the top. For genes with multiple probesets the most significant *P*-value is considered. Red bars indicate significance (*P*<0.05). Genes without a *P*-value were not detected on the array and thus not tested.

To assess the effect of the WBS microdeletion on genome-wide expression, we first profiled the transcriptome of primary skin fibroblasts of eight WBS patients and nine sex- and age-matched control individuals using Affymetrix expression arrays (see **Table S1** for the complete list of samples). These data have been deposited in the NCBI Gene Expression Omnibus under accession number GSE16715. Comparison of the WBS individuals with controls using moderated *t*-statistics revealed differentially expressed transcripts, including some of the hemizygous genes, thus partially confirming previous results [44] (see below). At a false discovery rate (FDR) of 0.05 we identified 1,114 probesets as differentially expressed, corresponding to 868 genes, which are listed in **Table S2**. (At a FDR of 0.01 we obtained 367 probesets, corresponding to 306 genes, see **Table S2**). All *P*-values shown were corrected for multiple hypotheses testing using the Benjamini-Hochberg method [51]. 56 HSA7 genes are differentially expressed, significantly more than expected by chance (Fisher's exact test, *P* = 0.032). Eight out of 13 monitored hemizygous genes were differentially expressed, again, more than expected by chance (Fisher's exact test, *P* = 6×10^−5^). Furthermore, 3 other out of the 13 hemizygous genes showed a trend towards downregulation, albeit not statistically significant (**Figure 1** and **Table S3**). These hemizygous genes, as a gene set, are underexpressed (gene set enrichment analysis, *P* = 0.0015). We note that, consistent with previous results, in particular our own analyses [44], microarrays detect a lower number of genes than quantitative PCR, due to their narrower dynamic range.

#### Enrichment analysis of the differentially expressed genes

We used these 868 differentially expressed genes (DEG) to perform gene enrichment analyses. A hypergeometric test on Gene Ontology (GO) categories uncovered a significant overrepresentation of extracellular matrix genes (*P* = 3.59×10^−5^) and class I major histocompatibility complex (MHC) genes, as well as genes the products of which localize to the postsynaptic membrane (all *P*<0.05, see **Table 1** for details). Closer examination of genes coding for extracellular compartment proteins revealed an overrepresentation of biological adhesion and binding, as well as structural molecules, while localization and transporter activity were underrepresented functions (**Figure S1**).

**Table 1:** GO terms enriched in the set of differentially expressed genes.

GO ID	BH-adjusted P-value	Direction	Odds Ratio	Expected Count	Count	Category Size	Term
GO:0005576	2.64E-06	ind/sup	2.12	48.61	89	592	*extracellular region*
GO:0031226	2.29E-05	ind/sup	2.26	32.19	63	392	*intrinsic to plasma membrane*
GO:0031012	3.59E-05	ind/sup	3.25	11.58	31	141	*extracellular matrix*
GO:0005887	3.70E-05	ind/sup	2.2	31.78	61	387	*integral to plasma membrane*
GO:0005578	6.27E-05	ind/sup	3.21	10.92	29	133	*proteinaceous extracellular matrix*
GO:0044421	1.01E-04	ind/sup	2.32	23.73	48	289	*extracellular region part*
GO:0044459	3.60E-04	ind/sup	1.75	57.32	90	698	*plasma membrane part*
GO:0005886	1.59E-03	ind/sup	1.52	101.58	139	1237	*plasma membrane*
GO:0042612	2.17E-03	ind	11.28	1.15	7	14	*MHC class I protein complex*
GO:0005581	8.08E-03	ind/sup	6.45	1.81	8	22	*collagen*
GO:0042611	8.08E-03	ind	7.9	1.4	7	17	*MHC protein complex*
GO:0044420	1.27E-02	ind/sup	3.51	4.52	13	55	*extracellular matrix part*
GO:0032393	1.82E-02	ind	22.11	0.75	6	9	*MHC class I receptor activity*
GO:0005201	1.82E-02	ind/sup	5.55	2.76	11	33	*extracellular matrix structural constituent*
GO:0045211	1.87E-02	ind/sup	4.62	2.55	9	31	*postsynaptic membrane*
GO:0002474	2.00E-02	ind	10.86	1.36	8	16	*antigen processing and presentation of peptide antigen via MHC class I*
GO:0048002	2.00E-02	ind	10.86	1.36	8	16	*antigen processing and presentation of peptide antigen*
GO:0004888	3.74E-02	ind/sup	2.16	17.64	34	211	*transmembrane receptor activity*

Instead of considering the expression levels of single genes, a more robust approach is to work with gene sets. One such method is gene set enrichment analysis [52]–[54], in which the aggregated expression level of a pre-defined group of genes is tested for difference between two biological states. Yet, the scope of enrichment analyses for genes in pre-defined functional categories is limited for several reasons: first, even though more than 80% of human genes have now been annotated in GO, the experimental evidence for these annotations differs widely (with less than 30% of the genes having at least one experimental annotation [55]). Second, the categorization and annotation is obviously biased by human interpretation and reflects research foci. Finally, co-regulation of genes belonging to a functional category may not be induced transcriptionally or if so, only partially. In order to overcome these limitations, we sought to complement our enrichment analysis with functional gene categories directly derived, in an unbiased manner, from gene expression data. We refer to such units of transcripts that exhibit coherent expression across a subset of the experimental samples as *transcription modules* (see below). This approach is based on the hypothesis that transcripts belonging to the same module are likely to play a role in the same pathway (or any biological process) and that their average expression levels can be used as a proxy for the induction or suppression of this pathway. An additional benefit of this approach is that it can also highlight novel functional links for genes that have no or fragmented annotation so far.

### Using modular analysis to explore the pathophysiology of WBS

#### Identifying transcription modules from fibroblast expression data

In our first modular study (to which we refer as **M1**), we collected skin fibroblast microarray datasets, unrelated to our study, and used them to identify sets of co-expressed genes in fibroblasts (see **Table S4** for a complete list of included datasets, their descriptions and accession numbers). Towards this end we used the Iterative Signature Algorithm (ISA) [56], a powerful tool for the rapid identification of transcription modules. Briefly, the ISA identifies, from a large set of expression data, subsets of samples for which certain sets of genes are coherently over- or underexpressed. We refer to these subsets as modules, and each sample and gene receives scores indicating their membership (if non-zero) and contribution to each module. The algorithm found 1'094 modules of genes that are co-expressed in specific subsets of samples. An interactive database of these modules is accessible online at http://www.unil.ch/cbg/ISA/Fibroblasts. They reflect the transcriptional responses to the given perturbations, either natural or specific to the experiments that were conducted on the fibroblast samples. 916 out of the 1'094 modules are functionally enriched, indicating that they correspond to co-regulated genes involved in particular pathways that are transcriptionally regulated.

To test whether some of the identified modules are differentially expressed in WBS patients compared to controls we calculated the weighted average expression of the genes of each module, using the ISA gene scores as weights. This was done separately for each WBS and control sample, after which the two groups were compared using a *t*-test. We identified 72 modules with significantly altered expression, by applying a 0.05 cutoff on the Benjamini-Hochberg corrected *P*-values (**Table S5**). A permutation test was used to validate these results (see Materials and Methods for details). The functional enrichments of these modules are consistent with those in the single-gene differential expression analysis. Indeed, many modules are enriched in genes annotated for the extracellular compartment and immune response, but also in DNA binding and transcription (a summary is given in **Table 2**, see **Tables S5** and **S6** and **Figure S1** for details).

**Table 2:** Summary of GO terms and KEGG pathways enriched in the dysregulated transcription modules, M1 modular analysis.

GO ID	BH-adjusted p-value	Count	Category size	Best module (size)	GO term
GO:0005576	2.92E-07	44	445	958 (294)	*extracellular region*
GO:0031012	5.20E-05	18	114	957 (323)	*extracellular matrix*
GO:0045449	1.23E-04	90	1084	1012 (542)	*regulation of transcription*
GO:0010468	1.23E-04	97	1213	1012 (542)	*regulation of gene expression*
GO:0005125	1.25E-04	7	34	349 (75)	*cytokine activity*
GO:0003677	1.25E-04	85	971	1012 (542)	*DNA binding*
GO:0032501	1.29E-04	30	1167	349 (75)	*multicellular organismal process*
GO:0009887	1.29E-04	14	224	349 (75)	*organ morphogenesis*
GO:0002376	1.58E-04	16	327	349 (75)	*immune system process*
GO:0042127	2.17E-04	15	291	349 (75)	*regulation of cell proliferation*
GO:0005057	3.02E-04	8	88	753 (120)	*receptor signaling protein activity*
GO:0009611	3.88E-04	11	156	349 (75)	*response to wounding*
GO:0042612	4.51E-04	6	13	1037 (341)	*MHC class I protein complex*
GO:0008283	5.55E-04	17	437	349 (75)	*cell proliferation*
GO:0006954	6.16E-04	10	98	747 (151)	*inflammatory response*
GO:0009605	6.69E-04	13	252	349 (75)	*response to external stimulus*
GO:0007165	8.14E-04	30	1342	349 (75)	*signal transduction*

#### Including the WBS data in the discovery of modules

**Figure 2:**
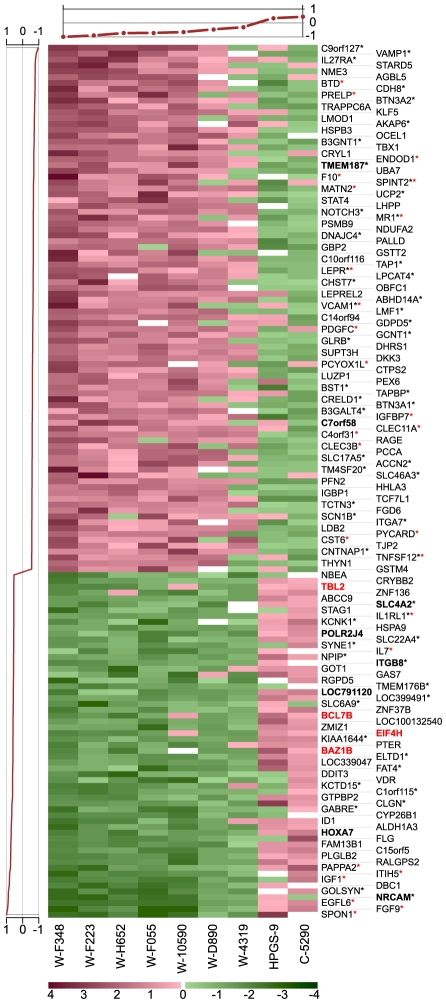
Example of a WBS dysregulated module (#770 from the M2 module set). This module contains 149 genes (one per line) and 9 samples (columns). Seven samples are from WBS patients (denoted with “W”), C-5290 is a control sample from our dataset, while HPGS-9 belongs to a publicly available dataset. Gene scores are plotted on the left and sample scores at the top. The 59 genes with positive gene scores (bottom lines) are downregulated (green) in the seven WBS samples and upregulated (red) in the other two. The remaining 90 genes show the opposite pattern: they are upregulated in the WBS samples and downregulated in the remaining two samples. Hemizygous gene names are emphasized in red and the names of genes mapping to HSA7 in boldface. Red asterisks indicate genes belonging to the GO category “extracellular region” while black asterisks denote genes from the “intrinsic to membrane” category.

Next, we searched specifically for coherent perturbations in gene expression driven by the WBS deletion. To this end, we performed a second modular study (to which we refer as **M2**), which included both the WBS samples and the data sets used previously. The ISA algorithm found 1,035 modules, of which 868 are functionally enriched and 368 contain at least one sample from our study. An interactive database of these modules is accessible online at http://www.unil.ch/cbg/ISA/Fibroblasts. Out of the 368 modules including one of our samples, 290 contain at least ten genes and were tested for differential expression. Specifically, a *t-*test, as above, on the weighted mean expression of these module genes identified 23 modules that were significantly dysregulated in the WBS case samples (listed in **Table S7**). An example of such a module is given in **Figure 2**. The remaining modules with unchanged expression thus represent functions that are unaffected in WBS. To check the significance of this result we randomly permuted the WBS case/control labels 1,000 times. We observed that none of these permutations yielded even a single dysregulated module.

#### Hierarchy of the modules

**Figure 3:**
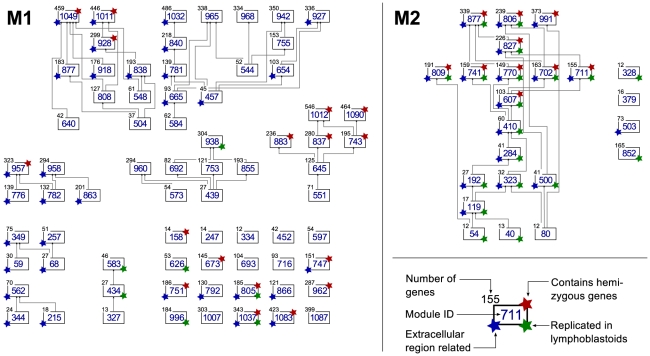
Hierarchical diagram of the transcription modules dysregulated in WBS identified in the M1 (left) and M2 (right) modular studies. Directed edges indicate direct subset relationships, and they always point upwards. The number of genes in a module is shown at the top left corner of the module box. Modules annotated with a red star on their top right corner contain at least one hemizygous (or flanking) gene; the ones with green stars on their bottom right corner were replicated in lymphoblastoid cell lines; blue stars on the bottom left corner indicate modules that show significant enrichment for extracellular region genes. An interactive version of this figure is available in the online supporting material at http://www.unil.ch/cbg/ISA/Fibroblasts, which allows to further query the gene content and functional enrichment of the modules.

Several smaller modules are included completely in other larger ones, forming a hierarchical structure. We organized the 72 and 23 dysregulated modules identified in M1 and M2, respectively, into a directed graph based on their subset relationships, i.e. two modules are connected by a directed edge, if all the genes in the first module are included in the second (see **Figure 3** and http://www.unil.ch/cbg/ISA/Fibroblasts). This graph has nine non-trivial components, with 3 to 19 modules each. Some of these modules can be readily linked to the WBS phenotype based on their functional enrichment, e.g. modules M1-349 and M1-257 (75 and 51 genes, respectively), which display multiple functional enrichments, notably in vasculature development and regulation, response to wounding, as well as chemotaxis and immune response (see website for the full lists and details). Interestingly, both modules contain the *NR4A3* gene (M1-349 also contains *SPRY2*), which are genes involved in the development of the inner ear. About one quarter of the gene products of these two modules localize to the extracellular region (19/75 and 14/51 genes, respectively).

#### WBS hemizygous genes in the dysregulated modules

We found that the dysregulated M1 modules include only two hemizygous genes (i.e. *WBSCR22B* and *LAT2 (a.k.a. WBSCR5)*), while five other hemizygous genes, namely *EIF4H*, *BAZ1B*, *BCL7B*, *ELN* and *TBL2*, were integrated into a total of 10 dysregulated M2 modules. All these genes, except *LAT2*, show differential expression between WBS case and control samples (see **Figure 1** and **Table S3**). Furthermore, among the 844 genes that compose the 23 dysregulated M2 modules, HSA7 genes are overrepresented, appearing 1.37 times more frequently than expected by chance (*P* = 0.048, Fisher's exact test). Modules containing hemizygous genes are enriched in membrane and extracellular proteins, as well as genes involved in immune response and organ development (a summary of the functional enrichment of M2 modules is given in **Table 3**, see **Tables S7** and **S8** and **Figure S1** for details).

**Table 3:** Summary of GO terms and KEGG pathways enriched in the dysregulated transcription modules, M2 modular analysis.

ID	BH-adjusted p-value	Count	Category size	Best module (size)	Term/name
GO:0005576	1.78E-12	73	700	991 (373)	*extracellular region*
GO:0006955	1.62E-06	17	295	503 (73)	*immune response*
GO:0031224	2.09E-05	89	2051	806 (239)	*intrinsic to membrane*
GO:0009605	2.78E-05	17	370	503 (73)	*response to external stimulus*
GO:0005102	1.02E-04	16	408	503 (73)	*receptor binding*
GO:0007165	3.95E-04	35	1800	503 (73)	*signal transduction*
GO:0042824	4.27E-04	4	6	702 (163)	*MHC class I peptide loading complex*
GO:0007154	7.32E-04	36	1947	503 (73)	*cell communication*
GO:0008083	8.75E-04	8	94	503 (73)	*growth factor activity*
GO:0042330	2.88E-03	7	69	503 (73)	*taxis*
GO:0009887	2.88E-03	13	312	503 (73)	*organ morphogenesis*
GO:0007626	2.98E-03	8	101	503 (73)	*locomotory behavior*
GO:0005578	3.21E-03	18	156	991 (373)	*proteinaceous extracellular matrix*
GO:0001525	3.47E-03	8	104	503 (73)	*Angiogenesis*
KEGG: 4060	7.51E-04	10	122	503 (73)	*Cytokine-cytokine interaction*

#### Genes that appear frequently in dysregulated modules

**Figure 4:**
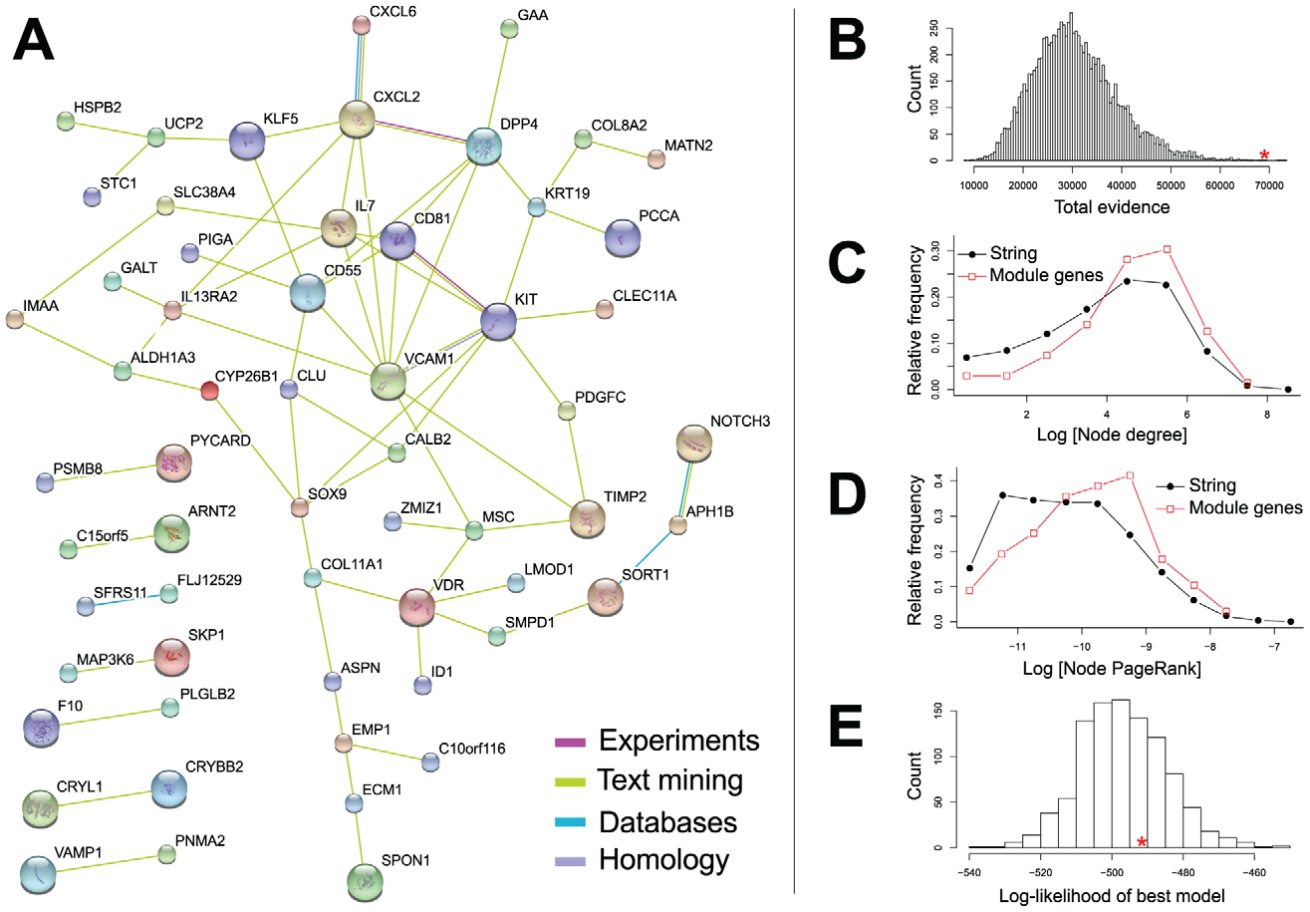
The network of the most frequent genes in the modules, as a subset of the STRING protein interaction database. Only genes that appear at least ten times in the dysregulated modules are considered. (**A**) Most frequent module genes that have at least one connection in the STRING database. Edges with evidence score higher than 0.3 are shown; their colors indicate different kinds of interaction evidence (key bottom right). (**B**) Most frequent module genes form a network that is denser than a random subnetwork of the same size in STRING. We generated 10,000 random subnetworks and calculated the sum of the evidence for all edges. Only five out of all random subnetworks show a higher total evidence value than the most frequent module genes indicated by a red asterisk (sum of total evidence = 69,033). (**C**) Distribution of the number of connections (node degree) per protein in the complete STRING network (black, filled circles), and the subnetwork of most frequent module genes (red, open squares). The subnetwork has significantly less low-degree nodes and more high-degree nodes (Wilcoxon-test *P* = 1.612×10^−5^). (**D**) Distribution of PageRank centrality scores in the complete STRING network and the subnetwork of most frequent module genes. The subnetwork has fewer non-central nodes and more central nodes (Wilcoxon-test *P* = 2.628×10^−5^). (**E**) We fitted hierarchical models [60] to the subnetwork of the most frequent module genes, and also to 1,000 randomized networks. The network of frequent module genes (red asterisk) shows no hierarchical structure compared to the randomized networks.

The severity of a phenotype correlates with the connectivity and thus centrality of the associated gene within the functional network [57], [58]. Based on this observation, we reasoned that the most frequent genes among our expression modules — and hence with the most connections in our dataset — are more likely to play a central role in the pathophysiology of WBS. We therefore considered the genes that were found by both the M1 and M2 modular studies and counted their occurrence in dysregulated modules. The M1 dysregulated modules contain 1984 different genes, while 844 different genes appear in M2 modules. 392 genes are present both in M1 and M2 modules, the most frequent ones being: *UCP2*, *EGFL6*, *C10orf116*, *HSPB2*, *PSMB9*, *SPON1*, *C4orf31*, *GABRE*, *ABHD14A* and *AGBL5* (see **Table S9** for a more complete list). The frequency of a gene in both module sets does not correlate with its differential expression for the first set of modules (M1, Pearson correlation 0.07), and it correlates positively for the second set (M2, Pearson correlation 0.33). To verify the functional connectivity of these most frequent genes we interrogated the STRING database that compiles known and predicted protein-protein interactions (http://string-db.org) [59]. We found that not only do these genes interact more with each other than expected by chance, as measured by the number of edges connecting them, but they also have more connections to the whole than a random subset of gene products. They also tend to have higher centrality scores and thus are closer to the center of the protein interaction network (**Figure 4A–D**). This correlation between frequency in the modules and degree of connectivity or centrality holds true for all genes in all modules regardless of their dysregulation in WBS (see **Figure S2**). To understand better the organization of the network of frequent genes, we fitted a hierarchical statistical model [60] to it. In this context, hierarchy means that the genes are organized into groups, within which they are connected with a higher probability. These groups are organized into even denser subgroups, and so on. The statistical model infers such a structure from the data. According to our results, however, the network of frequent genes lacks a hierarchical structure (**Figure 4E**). GO and KEGG enrichment calculation for the 392 common transcripts shows significant enrichment for several categories consistent with those identified in the single-gene differential expression analysis and the modules (**Table S10**).

Interestingly, the function of some of these frequently occurring genes may be relevant to the pathophysiology of some WBS features, such as metabolic phenotypes (*UCP2* [61]), dental anomalies (*SPON1* [62]), neurological features, cognition or brain development (*HSPB2*, [63], *ABHD14A* [64] and *GABRE* [65]). Also, the overrepresentation of genes related to the immune response in the list of most frequent genes hints at a putative immunological component of the syndrome, which has hitherto not been suspected from the clinical phenotype alone.

### Comparison with lymphoblastoid cell lines from WBS and control individuals

Gene expression in fibroblasts can only provide a partial picture of the gene dysregulation that gives rise to the WBS clinical phenotypes. Thus, data from other cell types or tissues may provide additional clues as to dysregulated pathways, as well as confirm some of our findings in fibroblasts. Indeed, comparison with the recently published transcriptome of lymphoblastoid, i.e. EBV-transformed, cell lines from WBS patients [66] revealed a few commonly dysregulated genes. The expression of 11 common genes was altered with the same sign in both cell types, while for 29 others we observe opposite expression (**Table S11**). Eight of the 11 genes with consistently altered expression were part of 28 dysregulated M1 or M2 modules (**Table S11**).

Out of the 72 M1 modules the average gene expression of which is altered in WBS fibroblasts, seven are also changed in the lymphoblastoid cell lines; four modules are altered in the same direction, three modules are opposite in the two studies. Moreover, 19 of the 23 dysregulated M2 modules are also perturbed in the lymphoblastoid samples, 18 in the same direction (**Table S11**), suggesting that the pathways identified in the fibroblasts are disrupted in multiple tissues. Furthermore, we can surmise that modules consistently regulated in both cell types may represent central pathways influenced by the WBS deletion, while the remaining modules may reflect cell-type specific alterations, which in turn might be important for tissue-specific phenotypes.

## Discussion

We have profiled the transcriptomes of skin fibroblasts from eight WBS patients and nine sex- and age-matched control individuals, and identified a number of transcription modules dysregulated in WBS patient cells. One caveat of this study lies in the use of isolated cells *in vitro* that may not reflect all the different tissue-dependent transcriptional changes *in vivo* that give rise to the complex WBS phenotypes, such as cognitive features or connective tissue anomalies. Moreover, the samples we consider only allow us to observe the downstream global effects of the primary cause, as opposed to the immediate effect on early development. However, these cell types are the most readily available samples, and the replication of a subset of the fibroblast dysregulations in lymphoblastoids supports the hypothesis that at least some of these changes appear in multiple cell types as a direct result of the 7q11.23 deletion and thus provide clues about pathways that may generally be perturbed in WBS. Our results reveal a transcriptional network which may contribute to the pathophysiology of WBS. We propose that many of the WBS phenotypes arise due to the dysregulation of a few key gene products, which influence (possibly in concert) “regulatory subnetworks”, leading to specific traits. Also, disturbances in a process due to one group of genes may trigger compensatory mechanisms in another set, either directly in the cell, or indirectly through intercellular or more systemic effects.

Both our single-gene and modular analyses provide a resource to enable a deeper exploration of the pathophysiology of WBS, which may lead to the discovery of potential novel functional interactions between their products. Our study further exemplifies how integration of transcription data unrelated to the studied condition can be used to complement annotation-dependent analyses. Indeed, the modular approach reduces the complexity of the expression data, allowing a more targeted assignment of functional categories to specific sets of co-regulated genes. Consistently, Turcan *et al.* recently used a similar methodology to identify groups of genes coherently regulated during cochlear development, which allowed them to pinpoint candidate genes for further study [67]. It is important to underline that further investigations and more data are needed to distinguish between biologically relevant associations of differentially regulated modules and spurious co-expression signals. Nevertheless, we think that the information generated by our study (and made available at http://www.unil.ch/cbg/ISA/Fibroblasts) provides a testable set of candidate pathways dysregulated in WBS and possibly involved in mediating the wide range of associated phenotypes.

## Materials and Methods

### Ethics statement

We have obtained the approval of the ethics committees of the University of Lausanne (reference number Protocol 123/06) and of the “Hospices Civils de Lyon” for this project. All patients provided written informed consent for the collection of samples and subsequent analysis.

### Sample population

Skin fibroblasts of 8 classical WBS and 9 control Caucasian female individuals aged between 3 and 8 years (see **Table S1** for details) and similar numbers of passages were obtained from the cell culture collections of the Centre de Biotechnologie Cellulaire, CBC Biotec, CRB-Hospices Civils de Lyon, Lyon, France. The respective presence and absence, as well as the extent of the deletion were ascertained by SybrGreen real-time quantitative PCR as previously described [26].

### Cell culture, RNA extraction and microarrays

Human skin fibroblasts were grown in HAM F-10, supplemented with 10% fetal bovine serum and 1% antibiotics (all Invitrogen). Total RNA was prepared using TriZOL Reagent (Invitrogen) and RNeasy Mini Columns (Qiagen) according to the manufacturers' instructions. The quality of all RNAs was assessed using an Agilent 2100 Bioanalyzer (Agilent Technologies) and used as a template for complementary DNA (cDNA) synthesis and biotinylated antisense cRNA preparation. The synthesis of cDNA and cRNA, labeling, hybridization and scanning of the samples were performed as described by Affymetrix (www.affymetrix.com). The cRNA samples were hybridized to GeneChip Human Genome U133 Plus 2.0 arrays (Affymetrix). The chips were washed, stained and scanned, according to the manufacturer's protocol.

### Accession number

The data of the 17 expression arrays produced for this report have been deposited in NCBIs Gene Expression Omnibus (GEO, http://www.ncbi.nlm.nih.gov/geo/) and are accessible through GEO Series accession number GSE16715.

### Single gene expression data analysis

Expression data analyses were performed using GNU R (version 2.9.2) [68] and the Bioconductor package (version 2.4) [69]. All R package versions are listed in **Table S12**. Low-level analysis and normalization were done using GCRMA. For differential expression analysis we filtered the probesets and kept only those present in at least six samples, according to the Affymetrix Present/Absent calls calculated with the affy R package. To reduce noise, we also removed probesets that do not map to an Entrez gene. 18,429 probesets, mapping to 10,570 genes were tested for differential expression, using the moderated *t-*statistics, as implemented in the limma R package. In addition to the significant *p-*value, we required a minimum of 50% change for declaring a gene differentially expressed. 1,114 probesets, corresponding to 868 genes were found differentially expressed at the 5% FDR level, 367 probesets, mapping to 306 genes at the 1% FDR level. The FDR was controlled using the Benjamini-Hochberg correction [51]. Gene set enrichment analysis of the WBS hemizygous genes was performed by comparing the mean *t-*statistics of these genes, for the WBS patients and the control individuals; the reference distribution for this was established by permuting the phenotype labels 10,000 times [70]. Gene Ontology and KEGG Pathway enrichment was calculated via a hypergeometric test, using the eisa and GOstats Bioconductor packages. The enrichment *P*-values were corrected using the Benjamini-Hochberg method for the number of categories tested.

### Modular analysis

A transcription module comprises a subset of genes that are co-expressed in a subset of conditions [56]. The Iterative Signature Algorithm (ISA) [71] is an unsupervised method to identify such modules. It starts from many random initial sets of genes (seeds) that typically converge to a set of potentially overlapping transcription modules. The ISA assigns a signed score to every gene of the module and every sample of the module (zero scores imply that the gene or sample is not included in the module). The further the gene/sample score is from zero, the stronger the association between the gene/sample and the rest of the module. Co-expressed genes of a module have the same sign, whereas opposite signs signal opposite expression. The scores of the samples are exactly the same as the weighted averages of the expression of the module genes, the weights being the scores of the genes. Sample scores can be extended to the samples that are not included in the module, by calculating the same weighted average of the module genes for them. These samples have (in absolute value) lower scores than the module samples, by definition. The extended sample scores can be used to test whether the genes of a module are differentially regulated in some samples. The aim is to identify dysregulated transcription modules containing genes that are differentially expressed in the cases compared to the control samples.

### Discovering transcription modules in data sets unrelated to WBS (M1)

In the first ISA run, we used skin fibroblast samples from seven experiments from public repositories, as well as collaborators of the AnEUploidy consortium (the latter can be obtained by contacting the consortium at http://www.aneuploidy.eu/) (**Table S4**). For each dataset we downloaded the raw data and normalized them separately with the GCRMA method. The non-common probesets were omitted and the normalized expression data were merged; the data set included 22,277 probesets and 96 samples. To reduce noise we removed probesets that were called “Present” in less than ten samples, using the standard Affymetrix Present/Absent calls. We also removed probesets that were not associated with any Entrez gene. In order to avoid a bias towards genes with multiple probesets we only kept the single probeset with the highest variance for those genes. The final dataset included 9,329 probesets.

We applied the ComBat batch correction algorithm [72] to minimize non-biological variation; we used the “disease status” of the samples as an additional covariate for the correction (column “disease status” in **Table S4**). The additional covariate ensures that we do not remove the signal associated with the different syndromes in the data sets, only the systematic experimental variation. We ran ISA as implemented in the eisa R package [73], with gene thresholds 2, 2.2, …, 4 and sample thresholds 1, 1.2, …, 2. The ISA identified 1,094 transcription modules.

For the identification of the dysregulated modules, we used the GCRMA normalized WBS data set. Probesets that were called “Present” in less than six samples were omitted from the analysis. We only considered the 7,447 probesets that were included both in this filtered WBS data set and the modular study.

732 modules that contained at least ten genes were tested for dysregulation. For the dysregulation test we standardized the WBS expression data for every gene separately. Standardization is an important step, since the test for dysregulation involves the average expression of the module genes. Specifically, to test a module, we calculated the weighted average expression of its genes, separately for each WBS sample. The weights were defined by the gene scores of the module. Then a *t-*test with unequal variance was performed for the WBS cases against controls. The *t-*test *P-*values were corrected with the Benjamini-Hochberg method. At the 5% FDR level 72 dysregulated modules were found.

To check the significance of finding 72 dysregulated modules, we permuted the WBS case/control labels 1,000 times and tested for dysregulation as before. These permutations serve as a null-model to estimate how many dysregulated modules could have resulted by chance. Only 14 permutations yielded at least one dysregulated module. Within these 14 cases, the mean number of dysregulated modules was 12.1, the median 1.5. The highest number of dysregulated modules found for a permutation was 58. We note that the three permutations that yielded multiple (false positive) WBS dysregulated modules had almost correct WBS case/control labels: only one pair was swapped.

Hypergeometric tests were used to calculate the functional enrichment of the 72 dysregulated modules, with Benjamini-Hochberg correction for the number of categories and the number of modules tested. The significance threshold was chosen as 0.05.

### Including the WBS data in the discovery of modules

The second modular study (**M2**) was performed almost identically, but this time the WBS samples were also included in the data set. The ISA was run on 9,460 probesets and 113 samples, using gene thresholds 2, 2.2, …, 4 and sample thresholds 1, 1.2, …, 2. The ISA found 1,035 modules, of which 290 contain at least ten genes and one sample from our study. These were tested for dysregulation using *t-*tests for the sample scores of the WBS cases vs. controls, identifying 23 modules that are differentially expressed. As an additional validation, we permutated the labels of the WBS samples 1,000 times; no permutation showed any dysregulated modules. Enrichment calculation for the dysregulated modules was done the same way as for the M1 modules, using Benjamini-Hochberg multiple testing correction for the number of categories and the number of modules tested, and a significance threshold of 0.05.

### The network of genes that frequently appear in dysregulated modules

We used version 8.3 of the STRING database to interrogate the genes that frequently appear in the dysregulated modules. All network measures were calculated using the igraph R package [74]. We fitted hierarchical models [60] to the subnetwork of frequent module genes, and also to 1,000 randomized networks. For fitting the hierarchical models, we only considered the largest connected component of the network, consisting of 90 proteins and 203 connections among them. The randomized networks had the same degree sequence as the original network, and they were produced using Monte-Carlo methods [75].

### Enrichment calculations for the extracellular region genes

The enrichment calculations for the extracellular region genes (**Figure S1**) were done using hypergeometric tests and the eisa and GOstats R packages. Only the second level terms in the “Biological process” and “Molecular function” ontologies were tested.

### Comparison of WBS lymphoblastoid cell lines and primary skin fibroblasts, transformed and non-transformed cells, respectively

To identify genes commonly dysregulated in cells from WBS patients identified in this study and in [66], which uses two-color arrays (GEO accession number GSE18188), we tested the lymphoblastoid samples for differentially expressed genes. We used the moderated *t-*statistics and a fold-change threshold of 1.5 and applied the Benjamini-Hochberg multiple testing correction method to identify 574 differentially expressed genes. Forty of these are common with the 868 differentially expressed genes we found in the fibroblast samples. To test the dysregulation of the fibroblast dysregulated modules in the lymphoblastoid samples, we calculated the weighted mean log fold change of the module genes for each lymphoblastoid array, where the gene scores of the modules were used as weights. Then we used a *t-*test to check whether the mean log fold change is significantly above or below zero, followed by the Benjamini-Hochberg multiple testing correction method.

### Online supporting material

The modules and related details are available at http://www.unil.ch/cbg/ISA/Fibroblasts. These web pages contain the summary of all M1 and M2 transcription modules and their GO/KEGG enrichment statistics. An interactive version of **Figure 3** is also included; this allows the exploration and annotation of the dysregulated modules, using various criteria. It is also possible to query the modules that contain a specific gene, or a list of genes. See the help page of the supplementary material for details. Additionally, the modules can be visualized interactively with the online version of ExpressionView [76].

### Annotation data and databases

The expression array annotation data were taken from the hgu133a2.db (version 2.2.11) and hgu133plus2.db (version 2.2.11) Bioconductor packages. The GO.db package (version 2.2.11) was used for the Gene Ontology and the KEGG.db package (version 2.2.11) for the KEGG pathway data.

Software packages are listed in **Table S12**.

## Supporting Information

Figure S1 Over- and under-representation of GO biological process and molecular function terms among “extracellular compartment” annotated genes of the DEG list and each set of dysregulated modules. Dark coloured bars denote significant enrichment/depletion. *P*-values (p) and odds ratios (o) are indicated. Terms marked in boldface display consistent direction of change in all sets and with significance in at least one set.
(2.61 MB EPS)

Figure S2 (A) Relationship between the number of times genes appear in transcription modules (M1, M2, or their union) and their number of connections in the STRING database. First row: genes were binned according to their frequency in modules, and the mean STRING degree of each bin is plotted. The line is the fit from the linear regression of STRING degree on frequency, the slope is always significant with a p-value less than 10^−9^. Second row: the mean (black) and median (blue) degree is plotted for the genes that appear at least a given number of times in the modules. In other words, the first point is the mean/median degree of all genes, the second data point is the mean/median degree of all genes that appear at least once in a module, etc. There is a clear correlation between the frequency in the modules and STRING degree. (B) Relationship between the number of times genes appear in modules and their PageRank centrality in the STRING network. The plots are essentially the same as in (A), but the PageRank centrality is plotted instead of degree. There is a clear correlation between the frequency in the modules and the centrality of the genes in the STRING network.
(1.14 MB EPS)

Table S1 Cell line information.
(0.02 MB XLS)

Table S2 Differentially expressed genes in WBS samples compared to controls.
(0.23 MB XLS)

Table S3 Differential expression of the WBS hemizygous and flanking genes.
(0.02 MB XLS)

Table S4 Datasets used for modular analysis.
(0.02 MB XLS)

Table S5 Dysregulated modules, M1.
(0.04 MB XLS)

Table S6 GO/KEGG term enrichment in dysregulated M1 modules.
(0.07 MB XLS)

Table S7 Dysregulated modules, M2.
(0.03 MB XLS)

Table S8 GO/KEGG term enrichment in dysregulated M2 modules.
(0.05 MB XLS)

Table S9 Most frequently occurring genes among dysregulated M1 and M2 modules.
(0.08 MB XLS)

Table S10 GO/KEGG term enrichment among genes common to both sets of dysregulated modules.
(0.04 MB XLS)

Table S11 Dysregulated single genes and modules common to fibroblasts and lymphoblastoid cell lines.
(0.03 MB XLS)

Table S12 Software packages used for the analysis.
(0.03 MB XLS)
